# Recent Advances in Processing-Induced Changes in the Structure, Techno-Functional Properties and Nutritional Quality of Animal- and Plant-Based Food Proteins

**DOI:** 10.3390/foods14050764

**Published:** 2025-02-24

**Authors:** Ruifen Li, Norbert Raak, Laura Roman

**Affiliations:** 1 Department of Food Science and CiFOOD Centre for Innovative Food Research, Aarhus University, Agro Food Park 48, 8200 Aarhus N, Denmark; ruifen@food.au.dk; 2 Department of Food Science, Ingredient and Dairy Technology, University of Copenhagen, Rolighedsvej 26, 1958 Frederiksberg C, Denmark; 3 Food Technology Division, Department of Agricultural Engineering, University of Valladolid, Av. Madrid 50, 34004 Palencia, Spain; lroman@uva.es

## 1. Introduction

Proteins are essential macronutrients in the human diet and key structural constituents of many food products. In response to global challenges, such as climate change, the lack of sustainability in food systems, and increasing food insecurity, a significant amount of research has focused on food proteins in recent years. Indeed, there is growing interest in finding and utilizing alternative proteins from more sustainable sources, including plant-based proteins from legumes [1,2], oilseeds [3,4], cereals [5], and nuts [6], as well as in further improving the quality of common animal-based proteins (particularly milk proteins). Processing—including protein extraction and isolation techniques [7] and the further conversion of protein ingredients into foods through thermal treatment, high-shear mixing, and homogenization, as well as enzymatic modifications and fermentation—has been shown to profoundly impact protein structure and techno-functional properties, such as solubility, emulsification, foaming, and gelation [8,9], as well as improve their digestibility [10]. These properties are essential for developing food products with desirable textural attributes and flavors and enhancing the nutritional profiles of protein-rich foods. Moreover, the relationship between protein structure and its processing-induced modification is increasingly being elucidated through advanced analytical techniques, such as spectroscopy [11], microscopy [12,13], and computational modeling [14].

The interplay between food processing in both animal- and plant-based foods and its impact on the structural, techno-functional, and nutritional properties of the proteins has attracted considerable attention because of its pivotal role in enhancing global food security and driving food innovation and health. The studies featured in this Special Issue cover a wide range of research advancements, ranging from exploring the heteroprotein complex coacervation mechanism (*contribution 7*); protein extraction and solubilization techniques using high-shear mixing (*contribution 3*), membrane filtration (*contribution 6*), ultrasound (*contribution 9*), and an industrially scalable wet-extraction process (*contribution 10*); the development of more sustainable and/or healthier plant-based foods, like fibrous extruded meat analogs (*contribution 1 & 5*) and plant beverages (*contribution 2*), and low-fat products using protein-based fat replacers (*contribution 11*); and the final step of improving protein digestibility (*contribution 8*) and finding nutritious plant-based foods to promote gut health (*contribution 4*).

This Special Issue offers critical insights on technological and nutritional applications of animal- and plant-based proteins, linked to the colloidal and structural characteristics of diverse protein ingredients, paramount for developing innovative and nutritious food products that meet both consumer and societal demands, offering more sustainable processing and functional protein ingredients while paving the way for this future research field.

## 2. Contributions to This Special Issue

This Special Issue features 11 publications including 10 research articles and 1 review, which can be categorized into four main research topics.

### 2.1. Protein Extraction and Its Effect on the Structure and Technological Properties of the Protein Ingredients

Special attention has been paid to alternative methods for extracting food proteins to enhance their functionality while preserving their native architectures. In this sense, Malterre et al. (*contribution 3*) explored the application of high-shear mixing to lentil protein isolates, reporting significant enhancements in the solubility, surface hydrophobicity, and colloidal stability of lentil protein as well as a reduced particle size compared to isolates not subjected to shear mixing. These authors showcased the potential of more effective and oriented processing techniques to enhance the techno-functional properties of underutilized plant proteins, which could further facilitate their suitable integration into food formulations (i.e., improved dispersion and solubilization within the food matrix). Similarly, Sert et al. (*contribution 9*) applied an innovative technique, namely, ultrasound-assisted alkaline extraction, to pumpkin seed proteins derived from oil press cakes. They found that this technique resulted in higher protein yields and improved the techno-functional properties of the protein isolates, like solubility, foaming capacity, and stability, and that the native state of the isolated protein was better preserved compared to isolates subjected to the normal alkaline extraction procedure. Meanwhile, Hansen et al. (*contribution 10*) developed a scalable wet-extraction process for producing pea protein isolates with better-preserved structural integrity and lower polymerization, bridging laboratory scale extraction with pilot plant processing of the pea protein ingredients for future industrial applications. These authors found that both mild alkaline solubilization combined with isoelectric precipitation and salt solubilization coupled with membrane filtration resulted in superior solubility, gelation, and emulsification properties of the pea protein compared to the commercial pea protein isolate. The authors demonstrated that double solubilization procedures at mild pH conditions can be used to replace single solubilization of pea proteins at high alkalinity.

Apart from plant protein ingredients, emphasis has also been placed on milk protein ingredients. Raak et al. (*contribution 6*) studied the effects of transmembrane pressure on the colloidal structure of casein micelles during microfiltration and diafiltration. Their findings contribute to gaining a better understanding of how processing conditions can modulate the functionality of milk protein concentrates through modifications of the supramolecular structure of proteins.

### 2.2. Innovations in More Sustainable and Healthier Foods

High-moisture extrusion is one of the most versatile food processing techniques for producing plant-based alternatives whose textural attributes resemble those of animal meat. In this regard, Ribeiro et al. (*contribution 1*) demonstrated how screw speed and barrel temperature during high-moisture extrusion influence the texture, protein structure, and nutritional quality of soy-based extrudates. The extrusion conditions influenced fibrousness, anisotropy, and the reduction of antinutritional factors, with higher processing temperatures resulting in improved tenderness and anisotropic structuring, a result also related to the modification of the protein secondary structure (i.e., increased inter- and intra-molecular aggregation). This work provides valuable insights into tailoring extrusion parameters to achieve meat analogs with optimal textural attributes. Furthermore, Snel et al. (*contribution 5*) examined the feasibility of re-extruding soy and pea protein isolates to reduce waste during production. Their work demonstrates that re-extrusion can maintain fibrous texture formation, indicating that previous processing history does not significantly influence texture and offering a sustainable approach to protein re-processing and reducing food waste. Moreover, Roland et al. (*contribution 2*) investigated the proteolysis and generation of free amino acids in plant-based drinks from several plant sources during storage. Their findings highlight the importance of understanding storage-induced changes to ensure product quality and stability, providing valuable insights for improving shelf-life.

To improve human dietary health and meet consumer demands for more nutritious options, the development of high-protein, low-fat food products with limited deterioration of textural attributes is in demand. Nourmohammadi et al. (*contribution 11*) reviewed the fabrication methods and fat-mimicking mechanisms of protein-based fat replacers. The latest findings reveal several promising fat replacers, which can be classified into four different types. The first one is protein isolates/concentrates, like the commonly used whey protein that benefits from its high compatibility with other dairy ingredients and a matching flavor profile. The second type is microparticles, such as soy protein or bovine serum albumin, which can provide a smooth and creamy mouthfeel. The third type is protein–polysaccharide hydrogels, especially those produced using widely used ionic polysaccharides like gum arabic and pectin. The last type is microgels, which are categorized into fragments of protein hydrogels, protein assembles, emulsified gel droplets, and protein–polysaccharide coacervates. These fat replacers can be fabricated based on their different types, using processing techniques such as thermal–mechanical treatment, anti-solvent precipitation, enzymatic hydrolysis, complexation, and emulsification. This comprehensive review provides a roadmap for developing sustainable fat replacers with minimal caloric impact without compromising texture.

### 2.3. Protein Digestibility and Nutritional Quality of Plant-Based Ingredients and Foods

Plant-based protein ingredients from walnuts have high nutritional value but are easily oxidized during processing or storage due to their high content of unsaturated fatty acids. Therefore, Zhao et al. (*contribution 8*) explored the impact of oxidative modification on the structure and digestibility of walnut protein isolates. They found that the dual role of oxidation could promote intestinal digestion while it caused gastric digestion resistance. With a focus on nutrition, Calva-Cruz et al. (*contribution 4*) conducted a pilot trial to evaluate the impact of popped amaranth, a protein-rich pseudocereal, on the gut microbiota of stunted children. Their findings suggested that popped amaranth can serve as a nutritious food that supports gut health and combats malnutrition.

### 2.4. Heteroprotein Complex Coacervation

Soussi Hachfi et al. (*contribution 7*) investigated the influence of ionic strength on the coacervation process between lactoferrin and β-lactoglobulin using direct mixing and desalting protocols. Their study provides new insights into the electrostatically driven mechanisms underlying heteroprotein complex formation. The potential application in food products could be the lactoferrin and β-lactoglobulin complex coacervates work as microcapsules for bioactive and texturing agents.

## 3. Conclusions

The 11 contributions collectively highlight the significant advancements in the research topics defined in this Special Issue, focusing on understanding the impact of food processing on the structure, functionality, and nutritional quality of animal- and plant-based proteins. The articles in the four groups outlined in this Editorial offer a roadmap for future innovation in the field of food proteins. The advancements in protein extraction and processing techniques open new paths for enhancing the functionality and utilization of plant-based protein ingredients and enable more viable large-scale applications for protein extraction while highly preserving protein nativeness. Furthermore, the optimization of the high-moisture extrusion processing and re-extrusion processes of soy and pea proteins demonstrate the potential to produce high-quality fibrous plant-based meat analogs while reducing waste production, aligning with sustainability goals. Protein-based fat replacers also provide a promising solution for developing high-protein, low-fat products that meet consumer demands without sacrificing texture. At the same time, studies on protein digestion, oxidative stability, and gut health further emphasize the nutritional benefits of plant-based foods. Research on heteroprotein complex coacervation could also offer new strategies for designing innovative food ingredients. Finally, we expect that this Special Issue will inspire further research and innovation, helping the food industry to translate these developments into real-world and scalable technological and nutritional solutions.

## Data Availability

No new data were created or analyzed in this study. Data sharing is not applicable to this article.
